# Conceptualizing rurality: The impact of definitions on the rural mortality penalty

**DOI:** 10.3389/fpubh.2022.1029196

**Published:** 2022-11-03

**Authors:** Wesley L. James, Claire Brindley, Christopher Purser, Michael Topping

**Affiliations:** ^1^Department of Sociology, Center for Community Research and Evaluation, University of Memphis, Memphis, TN, United States; ^2^Department of Politics, Justice, Law, and Philosophy, University of North Alabama, Florence, AL, United States; ^3^Department of Sociology, Center for Demography and Ecology, University of Wisconsin-Madison, Madison, WI, United States

**Keywords:** rural definitions, rural/urban, mortality, rural mortality penalty, health disparities, rural disadvantage, United States

## Abstract

**Background:**

In the U.S., inequality is widespread and still growing at nearly every level conceivable. This is vividly illustrated in the long-standing, well-documented inequalities in outcomes between rural and urban places in the U.S.; namely, the *rural mortality penalty* of disproportionately higher mortality rates in these areas. But what does the concept of “rural” capture and conjure? How we explain these geographic differences has spanned modes of place measurement and definitions. We employ three county-level rural-urban definitions to (1) analyze how spatially specific and robust rural disparities in mortality are and (2) identify whether mortality outcomes are dependent on different definitions.

**Methods:**

We compare place-based all-cause mortality rates using three typologies of “rural” from the literature to assess robustness of mortality rates across these rural and urban distinctions. Results show longitudinal all-cause mortality rate trends from 1968 to 2020 for various categories of urban and rural areas. We then apply this data to rural and urban geography to analyze the similarity in the distribution of spatial clusters and outliers in mortality using spatial autocorrelation methodologies.

**Results:**

The rural disadvantage in mortality is remarkably consistent regardless of which rural-urban classification scheme is utilized, suggesting the overall pattern of rural disadvantage is robust to any definition. Further, the spatial association between rurality and high rates of mortality is statistically significant.

**Conclusion:**

Different definitions yielding strongly similar results suggests robustness of rurality and consequential insights for actionable policy development and implementation.

## Introduction

The relationship between health and place has been prominently featured in public health research for decades. One of the primary indicators of place in the U.S. is rurality, as demonstrated by the overwhelming evidence to a geographic penalty in health outcomes: the *rural mortality penalty* (1–3). The rural mortality penalty is historically relevant, as it represents a departure from a decades-long trend of urban disadvantage in mortality. As large swaths of the U.S. population began concentrating in large cities, morbidity and mortality rates exceeded those in rural places because of population density, substandard living conditions, spread of contagious diseases, inadequate sewage disposal, and poor water quality (4). This lasted until the mid-1900s with a period of relative equal mortality patterns due to improvements in public health infrastructure, vaccinations, physical examinations, and health education (5). Beginning in the 1980s, a dramatic reversal in place-based mortality disproportionately affecting rural places occurred, and has widened ever since.

Despite the evidence of rural disparities in health and mortality, it is not without nuanced findings from past research. In recent decades, various findings have shown that perhaps the age-structure of the population is responsible for higher rural mortality rates (6), or higher rates of age-sex-race adjusted mortality happens in urban areas (7). In more recent years, a flood of research on the rural disadvantage has emerged, suggesting that however health outcomes are measured, the rural disadvantage appears prominent. An often-overlooked feature of this body of work is the ambiguity of the term “rural” itself. The present work seeks to address this ambiguity by directly testing the impact of the three major definitions of rurality on mortality rates. We seek clarity on the extent to which the definitions of “rural” matter, first by analyzing a dichotomized definition of rural-urban as a baseline, and secondly by analyzing intra-rural definitions of varying conceptualizations of rurality. Our analysis reveals how robust place-based mortality rates are, expanding the conversation on how to address underlying disparities with policy solutions aimed at reducing the substantial gaps in rural-urban health and overall spatial inequalities.

### Rural health disadvantage

Rural populations in the United States have long experienced worse health outcomes than major cities, and these patterns persist. Continuing experiential and statistical evidence of rural disadvantage is clear and overwhelming, from the opioid epidemic (8–11) to disproportionate mortality rates and life expectancy (4, 12–14), and even the disparate effects of multiple COVID-19 variants (15–18). The enduring gap in rural-urban mortality nationwide is especially concerning and is increasing each year (19). This phenomenon, known as the *rural mortality penalty* (RMP), is well documented and has identified tens of thousands of additional deaths compared to urban places (1–3). This inequality of outcomes is associated with numerous societal factors—rural populations typically have higher unemployment rates, percentages of poor and uninsured residents, and are more vulnerable than their urban counterparts to economic downturns due to more concentrated economic specialization, among many others (20–22). Despite this spatially anchored pattern of disparate outcomes, there remains a lack of clarity in what “rural” means. Rural is often conceptualized as simply “non-urban,” but rural America is far from a homogenous collection of places. Though researchers have established various definitions of rural, we explore how variations in classifications matter, notably in how we understand overall inequality.

Given that current policy development and resource distribution depends heavily on institutional definitions, it is critical to understand how much our knowledge of rural disadvantage reflects reality or is an artifact of varying conceptualization and operationalization of “rural.” Currently, the three major coding schemes are (1) Rural Urban Continuum Codes (RUCC), (2) Urban Influence Codes (UIC), and the National Center for Health Statistics (NCHS) Urban-Rural Classification Scheme for Counties. Using these conceptualizations, we conduct an examination of nationwide rural-urban mortality rates to determine whether varying definitions of “rural” produce the same level of rural mortality disadvantage.

### Rural definitions

Prior research has shown how mortality and morbidity rates vary across rural-urban classification schemes. For example, when applying RUCCs, UICs, and NCHS codes to rural counties in Texas, one study found considerable variation in colorectal cancer incidence and mortality rates depending on the code examined (23). Other studies have shown similar varying results based on rural-urban definitions in cancer (24), access to hospitals and physicians (25), and all-cause mortality (1, 26). However, another effort based on county found little difference (27). In response to such variation, researchers have urged continued work to better understand the importance of rural and urban definitions, particularly as it pertains to health research. In fact, there has been a call for a nationwide classification study of all rural counties to further clarify how outcomes vary depending on the codes used (28). We attempt to answer this call by examining the three major rural-urban taxonomies, as the utility of these schemes may vary across regions and specific research aims.

The RUCC, UIC, and NCHS coding schemes undergird much of the social sciences, public health, and demographic literature on place in the U.S. Below we describe each of them:

- Rural-Urban Continuum Codes: These codes differentiate counties by population size and adjacency to metro areas (29). Codes 1 through 3 are urban, with population ranging from <250,000 to more than 1,000,000 people. Codes 4 through 9 indicate rural counties. The even-numbered codes (4, 6, and 8) are adjacent to metro areas, whereas the odd numbered codes (5, 7, and 9) are not adjacent to metro areas. Codes 4 and 5 have populations of over 20,000 people; codes 6 and 7 have populations ranging from 2,500 to 19,999, and codes 8 and 9 have fewer than 2,500 people.
- Urban Influence Codes: This is a twelve-code classification system of counties based on population size for metro areas, size of the largest city/town, and adjacency to metro and micro areas (30). Codes 1 and 2 are urban, stratified based on the county having more or fewer than 1,000,000 people. Codes 3 through 12 are categorized as rural and divided into two classifications: micropolitan (codes 3, 5, and 8) and noncore (codes 4, 6, 7, 9, 10, 11, and 12). The micropolitan codes are divided according to adjacency to large metro areas (code 3), small metro areas (code 5), and non-adjacency to metro areas (code 8). Likewise, the noncore areas are divided according to their adjacency to large metro (code 4), small metro (codes 6 and 7) and micro areas (codes 9, 10, 11, and 12), and those with a population of 2,500 people or fewer.
- NCHS Rural-Urban Classification Scheme for Counties: Six categories underscore urban distinctions by differentiating between central and fringe counties of large metro areas (26). The most urban category comprises the central counties of large metropolitan areas, and the most rural categories are noncore, nonmetropolitan counties. This means NCHS has more metropolitan levels (four) than micropolitan (two), largely because about 85% of the population lives in metropolitan areas (31).

While the preceding operationalization of rural areas utilizes ecological measures (e.g., population size and density) of the construct, these strategies neglect other approaches to delineating rural areas. Scholars have employed multidimensional conceptualizations of “rural,” incorporating occupational and socio-cultural elements into their definitions (32). This invokes Weber's *verstehen*, as they find, among a sample of Pennsylvania residents, laypeople conceptualize rural as being comprised of socio-cultural/occupational elements, such as a fondness for agrarian lifestyles, love of the wilderness, and an active distaste for urban ideals. This is an example of the wide range of criteria used to describe, explain, and define rural. Another key detail is that the terms rural and urban are technically not the same as nonmetropolitan and metropolitan, even though they are often used interchangeably (33). In our work, for ease of interpretation, we use the terminology “rural” consistently as we refer to non-urban places.

Further, we emphasize that though the RUCC, UIC, and NCHS codes are each based on Office of Management and Budget's (OMB) delineations of metropolitan and non-metropolitan statistical areas, our analysis primarily hinges on disaggregating various levels of rurality in each scheme, rather than only the rural-urban binary common across the three. In particular, the classification schemes themselves have underlying differences beyond how they categorize different levels of urban and rural—their respective data sets show slight differences in total number of counties as well as the corresponding total populations, as well as data reliability for certain counties over the decades. This reality notwithstanding, the differing population counts are not a part of our analysis, though the overall rural-urban patterns and results are not changed in any meaningful way. We report these numbers to provide context of how many people (and %) live in rural and urban areas. In terms of the actual data provided from each classification scheme's website, we only utilize the codes themselves to attach to our county-specific mortality rates.

## Methods

We use three classification schemes - RUCC, UIC, and NCHS—to assess robustness of mortality rates across these rural and urban distinctions. To address the changing classification codes that happens over time as a county increase or decreases in population, we implement a floating definition to all three. (We also tested the graphs with floating definitions of rurality against some using a fixed definition, and we conclude that our results are robust to both methods of fixed and floating definitions. The results are remarkably similar to those using the fixed definition, in terms of the overall 53-year pattern of each rural-urban designation, and the relative difference between definitions.) We assign schemes to years on a decade-by-decade basis. We then analyze a rural-urban dichotomy, combining all urban and rural subcategories together within each of the three schemes. This serves as our baseline understanding of broad temporal rural-urban patterns, and we delve further into degrees of intra-rural variation. Our primary focus is to determine if the rural disadvantage in mortality is similar regardless of the definition of rural. Second, we explore similar rural sub-categories across classification schemes to assess the magnitude of difference in mortality. This analysis of intra-rural variation highlights the level of robustness in rural disadvantage at a more precise level of measurement.

We use data from the Centers for Disease Control and Prevention (CDC) Compressed Mortality File (CMF). The CMF is a national population database that provides county-level data on U.S. mortality history. It measures all deaths by cause, age, race, sex, county of residence, and other characteristics recorded on death certificates by International Classification of Disease (ICD) codes (34). The mortality rates are measured in five-year averages and are age-adjusted to the 2000 Standard Million, per 100,000 people. The five-year averages provide stability for low-population rural counties. We analyze age-adjusted, all-cause mortality rates covering a period of 53 years (1968–2020), calculating, and graphing ten, five-year-averaged time periods and one three-year averaged time period, to assess rural-urban-specific trend lines by each classification scheme. Time periods are 1968–72, 1973–77, 1978–82, 1983–87, 1988–92, 1993–97, 1998–2002, 2003–07, 2008–12, 2013–2017, and 2018–2020. All-cause mortality trends are assessed by aggregated (dichotomous rural-urban classifications) and non-aggregated (intra-rural classifications) rural-urban status for the three major coding schemes discussed above (RUCC, UIC, NCHS).

We assigned RUCC, NCHS, and UIC codes to each of the 3,100+ U.S. counties and merged with 1968–2020 all-cause mortality data based on Federal Information Processing Standard (FIPS) codes. We assign coding schemes to years on a decade-by-decade basis as follows:

- NCHS: 1990 codes for 1968–1999, 2006 codes for 2000–2009, and 2013 codes for 2010–2020.
- RUCC: 1974 codes for 1968–1979, 1983 codes for 1980–1989, 1993 codes for 1990–1999, 2003 codes for 2000–2009, and 2013 codes for 2010–2020.
- UIC: 1993 codes for 1968–1999, 2003 codes for 2000–2009, and 2013 codes for 2010–2020.

The following two analyses are of: (1) the robustness in dichotomized rural-urban definitions, and (2) robustness in subcategories of intra-rural definitions. The classification of corresponding RUCC, UIC, and NCHS codes are presented in Table 1. Fifty-three-year trends in mortality within and across classification schemes are presented in the following section.

**Table 1:** Number, percent, and population of counties by RUCC, NCHS, and UIC codes.

**Rural code**	**# of counties**	**% of counties**	**Total population**	**% of population**
**RUCC**				
4	214	6.9%	13,538,322	4.4%
5	89	2.9%	4,670,365	1.5%
6	593	19.1%	14,784,976	4.8%
7	425	13.7%	8,113,866	2.6%
8	219	7.0%	2,155,622	0.7%
9	408	13.1%	2,546,256	0.8%
**NCHS**				
5	637	20.5%	26,912,771	8.6%
6	1311	42.2%	18,854,621	6.1%
**UIC**				
3	130	4.2%	7,190,190	2.3%
4	149	4.8%	3,243,787	1.1%
5	242	7.8%	11,180,286	3.6%
6	344	11.1%	7,290,442	2.4%
7	161	5.2%	1,574,215	0.5%
8	265	8.5%	8,486,815	2.8%
9	184	5.9%	2,798,944	0.9%
10	187	6.0%	1,339,635	0.4%
11	115	3.7%	1,818,968	0.6%
12	171	5.5%	886,125	0.3%

An additional analysis combines measures of association with geographically anchored spatial visualization techniques. We examined each grouping's data for spatial autocorrelation to test against the null hypothesis of spatial randomness (in which any mortality level is equally likely at any location), using global Moran's I (initial test for any patterns in mortality) and then local bivariate Moran's I for regional clusters (both rural and high mortality or both urban and low mortality) and outliers (rural and high mortality surrounded by the opposite or urban and low mortality surrounded by the opposite). Number and relation of neighbors was calculated using queen contiguity for spatial weights. The global Moran's I indicates the direction and magnitude of the spatial relationship between rurality and mortality, in the form of a coefficient between −1 and 1, along with a *p*-value. We then used bivariate local Moran's I to test significance of clusters and outliers in the relationship between rurality and mortality rates, comparing contiguous counties. This mapped the level of each county's spatial autocorrelation in terms of mortality to visualize statistically significant regional clustering of high rurality and high mortality rate, along with the opposite combinations thereof. The resulting maps illustrate the magnitude of association between mortality and each rural-urban coding scheme.

## Results

The number, percent, and population of counties by RUCC, UIC, and NCHS codes using the downloadable data is shown in Table 1. From this, there are 3,109 contiguous counties common to all three coding schemes, with some disqualified because they did not have a code in at least one of the schemes or because their population data were unreliable. (When merging in the all-cause mortality data, the number of contiguous counties moves to 3,081 due to some of the counties' all-cause mortality rates being unreliable.) Of the 3,109 total counties by population, 1,161 (37.3%) are classified as urban across all three coding schemes. Rural counties make up the remaining 1,948 counties (62.7%). Although nearly two-thirds of counties are rural, only 15% of the American population resides there. The other 85% of the population live in urban counties (15).

An investigation into rural sub-categories provides insight into the heterogeneity that exists in rural places. For instance, RUCC 6, which are counties adjacent to metro areas with populations of 2,500 to 19,999, are the most common rural places (*N* = 593) in the United States. Nearly 15 million people reside in these counties, accounting for 4.8% of the total population. Alternatively, the stereotypical characterization of rural places, e.g., RUCC 9, remote counties with population below 2,500, are less common (*N* = 408). These remote locations are occupied by only 2.5 million people, comprising <1% of the total population. According to NCHS codes, there are roughly twice as many counties that classify as neither micropolitan nor metropolitan (NCHS 6) than there are micropolitan counties (NCHS 5). However, there are 8 million fewer people in NCHS 6 compared to NCHS 5. With UIC codes, the modal number of counties classified as rural are those with the code 6 classification (noncore areas adjacent to metro areas, with populations of at least 2,500). UIC 6 contains 7.3 million people and only 2.4% of the total population. These figures demonstrate a few examples of the variation in rural conceptualization which may affect the results of spatially oriented statistical analyses.

**Figure 1:**
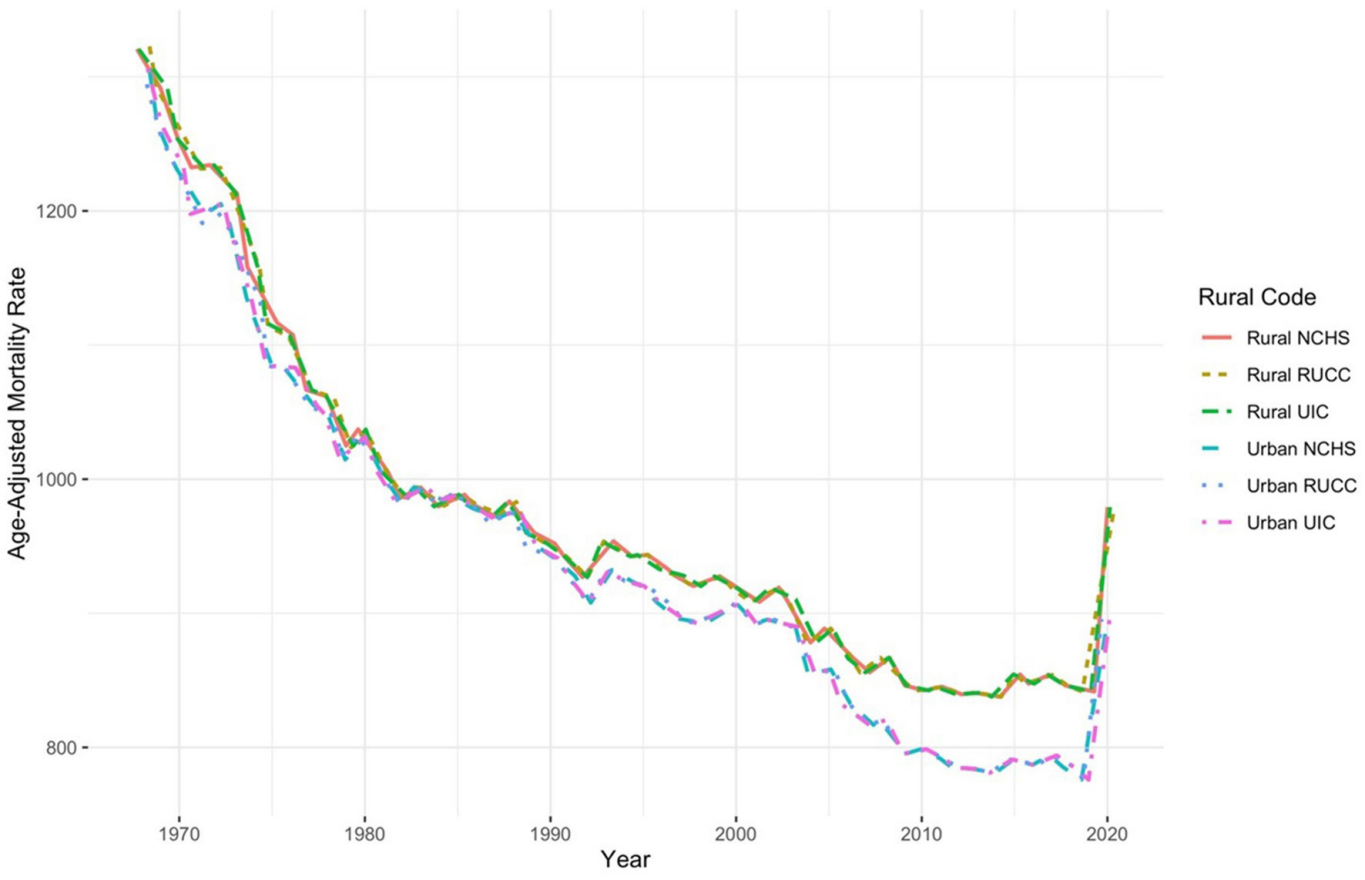
Dichotomized urban-rural comparisons of all-cause mortality, 1968–2020.

Figure 1 graphs comparisons of all-cause mortality between dichotomized urban and rural areas for the entire U.S. population from 1968 to 2020, for RUCC, UIC, and NCHS codes. The general trendlines are remarkably similar. All three urban definitions track precisely with one another throughout the time series, as do the three rural definitions. After nearly 20 years of no discernable rural-urban disparity, the rural disadvantage in mortality emerges in the mid-1980s and continues to grow through 2020, regardless of which rural-urban classification scheme is utilized, suggesting the overall pattern of rural disadvantage is robust to any definition. Pre-COVID, rural places exhibited a mortality rate of roughly 840 deaths per 100,000 compared to the urban rate of about 776 per 100,000. Another key observation consistent across classification schemes is the spike in mortality in rural and urban places in the last 2 years. This is largely the influence of COVID-19 substantially increasing mortality rates throughout the nation (35).

**Figure 2:**
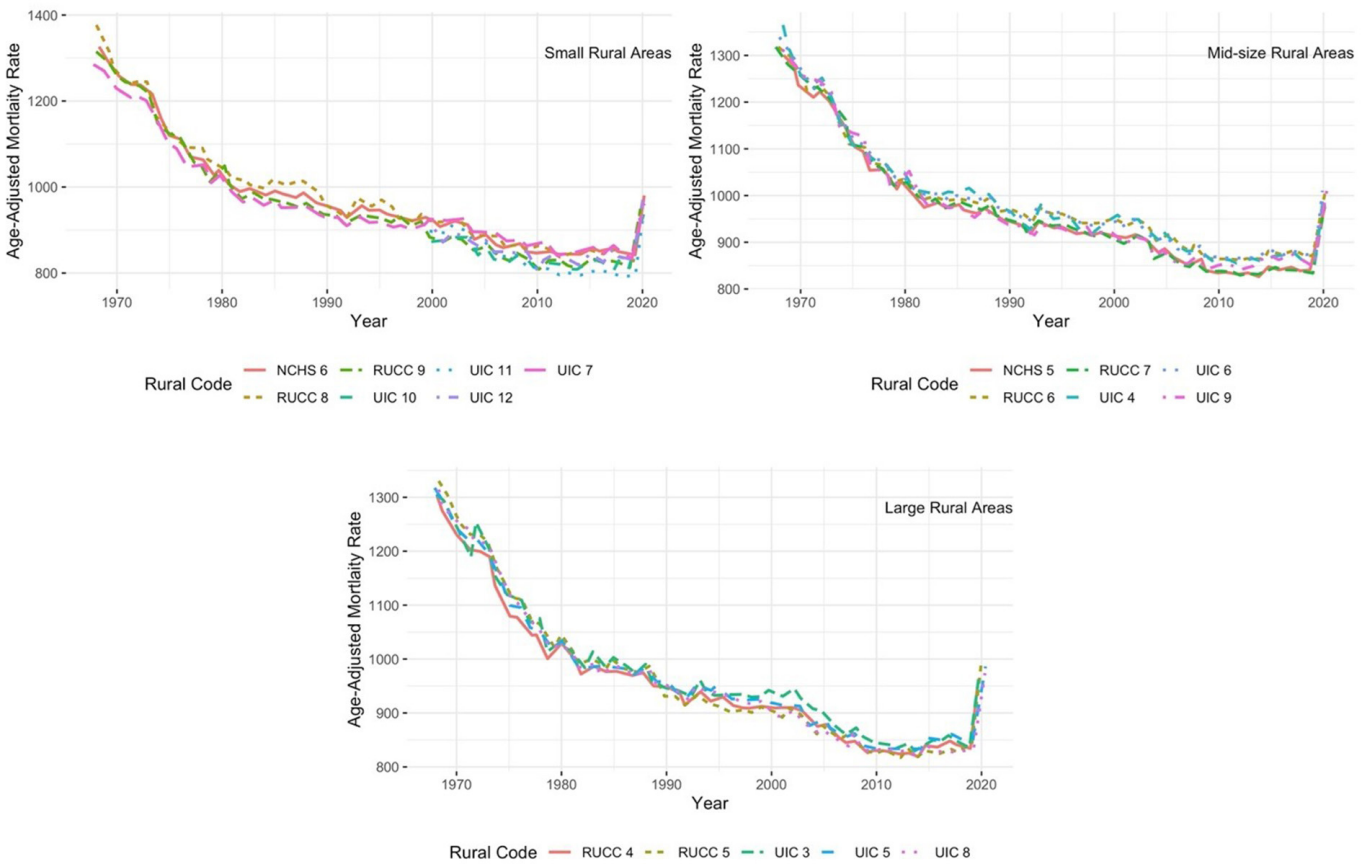
Intra-rural comparisons of all-cause mortality, 1968–2020.

Figure 2 focuses exclusively on rural subcategories across RUCC, UIC, and NCHS schemes for small, mid-size, and large areas. The categories for each classification scheme are as follows: (1) small rural areas: RUCC codes 8 and 9, UIC codes 7, 10, 11, and 12; NCHS code 6; (2) midsize rural areas: RUCC codes 6 and 7, UIC codes 4, 6, and 9; NCHS code 5; and (3) large rural areas: RUCC codes 4 and 5 and UIC codes 3, 5, and 8. Overall, the definitions follow a similar trend through the decades. In small rural areas, NCHS 6 counties (classified as noncore, nonmetropolitan counties) reliably exhibit higher levels of mortality (about 987 per 100,000 in 2020) than any other category but track very closely with UIC 12 (970 per 100,000) counties (noncore not adjacent to metro- or micropolitan area) and UIC 7 (961 per 100,000) (noncore adjacent to small metro). The other four categories (RUCC 8-9 and UIC 10-11) show slightly lower levels of mortality than their other rural counterparts. On average, the combination of small rural areas exhibits a collective mortality rate just shy of 956 deaths per 100,000. For mid-size, UIC 6 and 9 (about 1,010 per 100,000 in 2020) and RUCC 6 (1,009 per 100,000) show consistently higher mortality levels than RUCC 7, NCHS 5, and UIC 4. The collective mortality rate of mid-sized rural places is near 996 deaths per 100,000. And in large rural areas, mortality trends are the most similar across time; however, RUCC 5 (about 990 per 100,000 in 2020) and UIC 3 (948 per 100,000) are generally the highest and lowest, respectively. The collective mortality rate for these places is between their small and mid-size counterparts, hovering just above 974 deaths per 100,000.

**Figure 3:**
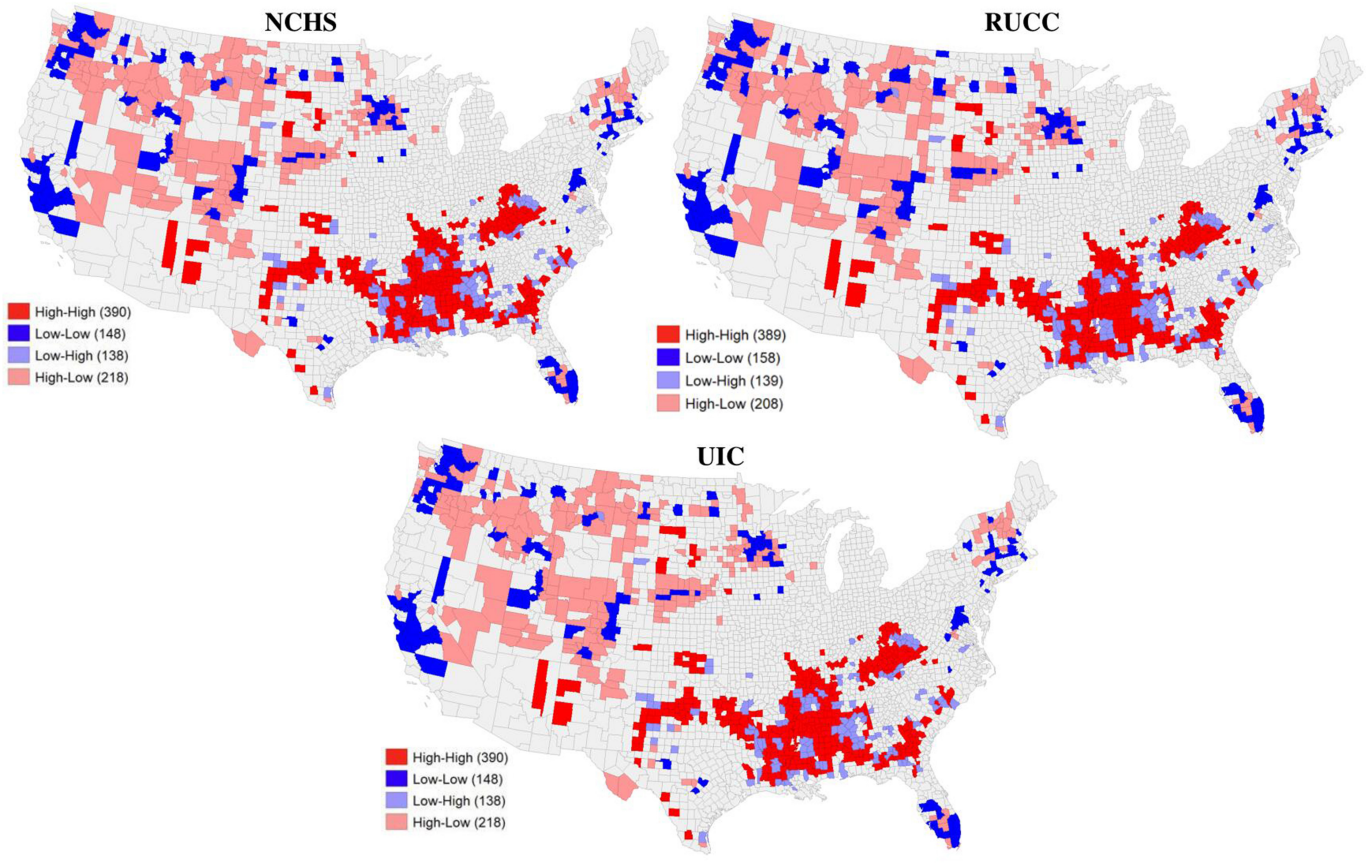
Spatial clusters of rurality and high age-adjusted mortality rates (all codes).

Finally, analysis of spatial clusters and outliers in rural-urban mortality rates show statistically significant concentrations of high mortality across the rural classification schemes, especially in the Southeast and Appalachia. An initial global Moran's I test for concentrations of high and low age-adjusted mortality rates shows positive autocorrelation—the mortality data does spatially cluster (I ≈ 0.5). Then we add rurality: looking at local Moran's I for a bivariate model (rurality and mortality) of spatial clusters and outliers. Figure 3 shows a statistically significant pattern to the clustering of mortality rates with rurality (*p* < 0.05). Bright red areas indicate county clusters that are both rural and are surrounded by counties with high mortality—with two-and-a-half times as many counties as the next cluster (bright blue areas: urban county areas surrounded by low death rates; gray indicates lack of significance). Being rural had a positive significant spatial correlation with high mortality rates, especially in Appalachia and the Southeast, which is consistent with the results of mortality alone. The rural spatial outliers (light red) reveal more counties with high mortality surrounded by urban spaces. Light-blue counties have low mortality but are surrounded by rural areas. Overall, Between the three classification schemes, similar numbers of counties are both rural and nested among high age-adjusted mortality rates.

## Discussion

The association between rurality and mortality is consistent across the three major classification schemes. This is not to argue that how “rural” is defined does not matter unilaterally—no single definition could ever capture the diversity of rural spaces and the combined interdependence and heterogeneity between places. But when examining rural-urban all-cause mortality disparities, the RMP persists across classification schemes, not only when conceptualized as dichotomized rural or urban, but also to a very large degree with smaller rural sub-classifications. To this point, researchers have argued that dichotomized rural-urban definitions mask variability within rural areas (28). However, our relatively unique finding is that the standard rural subcategories, e.g., intra-rural classifications, offer a large degree of uniformity in mortality outcomes.

The conceptualizations of rural-urban are secondary to rural disadvantage, which is robust across definitions. This fits social theorist Emile Durkheim's “social facts”—these definitions transcend the individual while constraining the individual (36). This reflects the persistence of place-based mortality and social determinants, spatial inequalities of access and outcomes, and a continued basis for further disaggregation of data and analysis of specific forces affecting different groups of rural residents.

Social determinants of mortality—poverty, education, income, race, etc.—are more concentrated in rural areas. As rural communities are left further behind with each passing generation, the level of relative deprivation compared to urban places continues to grow (37, 38). This stark inequality is the culmination of the many disparities apparent in American life. That the RMP is significant and still growing illuminates the gap between health improvements in urban and rural places. Mortality in rural places is indeed improving, but at a much slower rate than their urban counterparts (39), once again leaving rural Americans further behind. And as the COVID-19 pandemic has vividly shown, inequity in health is a collective, not individual, problem. Focusing on why these disparities exist and persist is a more powerful tool for solving them than centering the rural-urban definitional divide itself. Even where myriad inter-area variations may appear, the rural disadvantage still begets higher mortality.

Inequality is a destabilizing force (40), and grips the U.S. map from coast to coast. Discussion of its alleviation is incomplete without mention of space and the distribution of resources across places. Local, state, and national policymakers can use more targeted analysis to address imbalances in investment and facilitate equitable environments. Solutions are likely multi-faceted; for instance, policy change can address the scope of practice for nurse practitioners to enhance their ability to practice medicine independently, especially in places with a shortage of family doctors and specialists (41). State governments can attack problems through investment in struggling areas, a recent example being how ARPA funds are distributed by state and local governments (42). Lastly, local-level change can happen through program intervention aimed at addressing chronic disease in struggling areas through the implementation of telemedicine or other programs (43). Some of these policies and programs could be based on high mortality rates by place, rather than rural-urban classifications. Investing in both people and places better echoes the multidimensionality of this country's geography.

## Data availability statement

Publicly available datasets were analyzed in this study. This data can be found in the following repositories: United States Department of Health and Human Services (US DHHS), Centers for Disease Control and Prevention (CDC), National Center for Health Statistics (NCHS), Compressed Mortality File (CMF) on CDC WONDER Online Database (1968–78, 1979–1998, 1999–2020); 2020 U.S. Census Bureau, Cartographic Boundary Files.

## Author contributions

WJ is primarily responsible for the research idea, conceptualizing the data analysis strategy, and drafting the manuscript. CP conducted the literature review. CB contributed to the data analysis and constructed the graphs, table, and maps. MT conducted initial data analysis and constructed a set of graphs. All authors contributed to the article and approved the submitted version.

## Conflict of interest

The authors declare that the research was conducted in the absence of any commercial or financial relationships that could be construed as a potential conflict of interest.

## Publisher's note

All claims expressed in this article are solely those of the authors and do not necessarily represent those of their affiliated organizations, or those of the publisher, the editors and the reviewers. Any product that may be evaluated in this article, or claim that may be made by its manufacturer, is not guaranteed or endorsed by the publisher.
